# Plasmonic Contact Lenses Based on Silver Nanoparticles for Blue Light Protection

**DOI:** 10.1021/acsanm.3c05857

**Published:** 2024-03-06

**Authors:** Mohamed Elsherif, Ahmed E. Salih, Fahad Alam, Ali K. Yetisen, Khalil B. Ramadi, Haider Butt

**Affiliations:** † Department of Mechanical Engineering, Khalifa University, Abu Dhabi 17788, UAE; ‡ Division of Engineering, New York University Abu Dhabi, Abu Dhabi 129188, UAE; § Department of Chemical Engineering, Imperial College London, London SW7 2AZ, U.K.; ∥ Tandon School of Engineering, New York University, New York, New York 11201, United States

**Keywords:** contact lenses, biomaterials, blue light filtering, silver nanoparticles, pHEMA

## Abstract

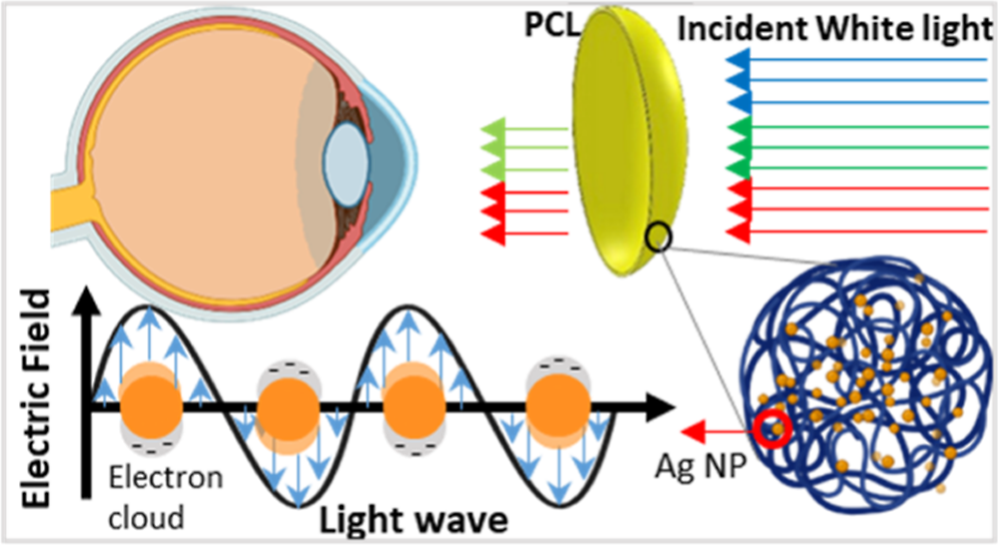

Constant exposure to blue light emanating from screens, lamps, digital devices, or other artificial sources at night can suppress melatonin secretion, potentially compromising both sleep quality and overall health. Daytime exposure to elevated levels of blue light can also lead to permanent damage to the eyes. Here, we have developed blue light protective plasmonic contact lenses (PCLs) to mitigate blue light exposure. Crafted from poly(hydroxyethyl methacrylate) (pHEMA) and infused with silver nanoparticles, these contact lenses serve as a protective barrier to filter blue light. Leveraging the plasmonic properties of silver nanoparticles, the lenses effectively filtered out the undesirable blue light (400–510 nm), demonstrating substantial protection (22–71%) while maintaining high transparency (80–96%) for the desirable light (511–780 nm). The maximum protection level reaches a peak of 79% at 455 nm, aligned with the emission peak for the blue light sourced from LEDs in consumer displays. The presence of silver nanoparticles was found to have an insignificant impact on the water content of the developed contact lenses. The lenses maintained high water retention levels within the range of 50–70 wt %, comparable to commercial contact lenses. The optical performance of the developed lenses remains unaffected in both artificial tears and contact lens storage solution over a month with no detected leakage of the nanoparticles. Additionally, the MTT assay confirmed that the lenses were biocompatible and noncytotoxic, maintaining cell viability at over 85% after 24 h of incubation. These lenses could be a potential solution to protect against the most intense wavelengths emitted by consumer displays and offer a remedy to counteract the deleterious effects of prolonged blue light exposure.

## Introduction

Consistent exposure to electronic devices such as smartphones, computer screens, and televisions has become a daily routine. However, the blue light emitted from these devices negatively impacts the user’s overall health, particularly their eyes.^1−5^ Blue light, with the shortest wavelength and highest energy band (400–515 nm) in the visible light spectrum (400–780 nm), has recently garnered attention due to its harmful consequences after prolonged eye exposure.^6^ This exposure has been linked to retinal damage, macular degeneration, lesions, and night blindness.^7−12^ Blue light may cause eye tiredness and soreness, and it can alter the photochemistry of the eye retina with detrimental effects.^13,14^

Notably, blue light suppresses the production of melatonin, the hormone responsible for sleep, resulting in insomnia or sleep deprivation. Our circadian rhythm and sleep–wake cycle are significantly influenced by light exposure. In the evening, the absence of blue light regularly found in sunlight prompts the pineal gland to produce melatonin, signaling the body to fall asleep. However, exposure to artificial light sources prevents sufficient melatonin secretion, reducing melatonin levels. Avoiding blue light in the evening can increase natural melatonin, but it is challenging in our connected and digitalized world.

While tinted spectacles or eyeglass lenses (“sunglasses”) equipped with blue light filters aim to reduce blue light reaching the eyes, they have limitations for certain outdoor activities, such as sports, where frames may limit peripheral viewing. Additionally, wearing sunglasses at a distance from the eyes can allow high-intensity blue light around the lenses to reach the eyes, causing contrast aberration and other vision conditions. Attempts to reduce scattered blue light may limit the field of view.

Soft contact lenses are widely used for vision correction and cosmetic purposes. While blue light protective contact lenses (BLCLs) based on nanoparticles like silicon dioxide and titanium dioxide have been reported, their low protection levels (20%) and narrow blockage bands limit their performance.^15,16^ Zinc oxide/cyclized polyacrylonitrile composite contact lenses were also reported for blue light protection, but they exhibited low transmittance (approximately 60%) in the passband, making them less suitable for indoor activities.^17^ Recently released commercial BLCLs rely on chemical dyes, which may degrade and leach with a potential biological toxicity. To our knowledge, neither commercially available BLCLs nor previously reported ones provide maximum protection at the 455 nm wavelength, crucial for safeguarding against blue light from artificial sources and digital devices.

Silver nanoparticles (AgNPs) undergo significant alterations in their physical, chemical, and biological attributes owing to their surface-to-volume ratio.^18^ This distinctive feature has resulted in their widespread application in various sectors. Notably, these nanoparticles serve as an antibacterial agent, contributing to industries such as manufacturing, household products, healthcare, optical sensors, cosmetics, pharmaceuticals, and the food industry.^19^ They play a vital role in diagnostics, orthopedics, and drug delivery.^4,20^ Recently, silver nanoparticles have gained prominence in textiles, wound dressings, and biomedical devices.^21−23^ The biological activity of AgNPs is influenced by factors such as surface chemistry, size, size distribution, shape, particle morphology, composition, coating/capping, agglomeration, dissolution rate, and particle reactivity in solution.^23^

Addressing the need to prevent harm from prolonged blue light exposure without restricting indoor and outdoor activities, we introduced plasmonic contact lenses (PCLs) designed to protect eyes from blue light. Silver nanoparticles were dispersed in the contact lens’s precursor gel and immobilized through photopolymerization. The optical performance of the developed contact lenses was tested and compared to that of commercial blue light protective spectacles. The stability of the performance was examined using artificial tears and a storage solution for one month. Finally, the water retention and cytotoxicity of the developed contact lenses were evaluated.

## Materials and Methods

### Materials

Ethylene glycol dimethacrylate (EGDMA) (98%), 2-hydroxyethyl methacrylate (HEMA) (97%), 2-hydroxy-2-methylpropiophenone (HMPP) (97%), dimethyl sulfoxide (DMSO), 3-(4,5-dimethylthiazol-2-yl)-2,5-diphenyltetrazolium bromide, and silver nanoparticles suspended in aqueous solution (0.02 wt %) were purchased from Sigma-Aldrich and used without any further purification. Raw 264.7 cells were purchased from Manassas, USA., Dulbecco’s modified Eagle’s medium (DMEM) containing 4.5 g/L d-glucose, 0.584 g/L l-glutamine, 0.11 g/L sodium pyruvate, fetal bovine serum (FBS), and penicillin–streptomycin were purchased from Fisher Scientific.

### Preparation of the Plasmonic Contact Lenses

Contact lenses of various silver nanoparticle concentrations were fabricated according to the following recipe. Briefly, a mixture of HEMA, EGDMA, and HMPP was created with a ratio of 100:1:0.5 vol %, respectively. Subsequently, three concentrations (low, medium, and high) were derived from a 40 nm stock-suspended nanoparticle solution with concentrations of 0.2, 0.4, and 0.6 wt %, respectively. Similarly, low, medium, and high concentrations of 60 nm silver nanoparticles (0.15, 0.35, and 0.7 wt %, respectively) were prepared from their respective stock solution. Each nanoparticle concentration (20 μL) was combined with the monomer solution (130 μL) to form the gel for the PCLs. The gels underwent a 20 min sonication process to ensure uniform nanoparticle distribution and prevent aggregation. Subsequently, the prepared gels were injected into contact lens molds and polymerized under UV light with a wavelength of 365 nm (UVP Cross-linker CL-1000L, Analytik Jena) for 10 min. The polymerized lenses were washed in a solution of ethanol and deionized (DI) water (50 vol %) to eliminate any unpolymerized residues, followed by a final rinse in DI water.

### Nanoparticles Characterization

The optical densities of the suspended nanoparticles with diameters of 40 and 60 nm were determined by a UV–vis spectrophotometer (USB 2000+, Ocean Optics). Morphology and size distribution analysis of the nanoparticles were conducted by employing a transmission electron microscope (Tecnai, 200 kV, resolution: 0.24 nm). A 300-mesh copper grid obtained from Ted Pella served as the holder for the nanoparticles. Subsequently, 10 μL of the nanoparticle solution was applied to the grid and allowed to dry in an oven at 50 °C for 2 h. This process was repeated three times to ensure a substantial deposition of nanoparticles on the mesh grid. Transmission electron microscopy (TEM) images were subjected to analysis using ImageJ to extract the nanoparticles’ diameters and distribution.

### Characterization of the Plasmonic Contact Lenses

The optical performance of the PCLs was examined by measuring transmittance using a UV–vis spectrophotometer (USB 2000+, Ocean Optics) coupled with an optical microscope (Zeiss, 20× lens). Transmittance readings were taken at three distinct sites within the contact lens’s central zone to assess the nanoparticle distribution within the hydrogel matrix.

A scanning electron microscope (FEI Nova NanoSEM 650, resolution: 0.8 nm) was employed to scrutinize the aggregation and distribution of nanoparticles within the PCLs. Prior to scanning electron microscopy (SEM) imaging, the PCLs underwent a drying process at 40 °C for 6 h and were subsequently sheared using a cutter. To prevent discharging during SEM imaging, cross sections were coated with a 10 nm thick palladium film.

The water content of the PCLs was determined by subjecting them to drying in an oven at 60 °C for 2 h, followed by recording the weight. Subsequently, the samples were immersed in DI water, with weights recorded every 2 h for 24 h until full hydration was achieved.

To evaluate the stability of the developed lenses, the samples were exposed to the contact lens storage solution and artificial tears for a duration of 4–2 weeks in each solution, separately. Transmittance measurements were taken weekly to monitor any potential regression in the optical performance attributed to nanoparticle leakage.

### Cytotoxicity Test for the PCLs

The cytotoxicity was assessed using the MTT (3-(4,5-dimethylthiazol-2-yl)-2,5-diphenyltetrazolium bromide) reduction assay with RAW 264.7 cells as models. In this assay, cells were seeded in 12-well plates at a concentration of 5 × 10^5^ cells mL^–1^ and incubated for 24 h at 37 °C and 5% CO_2_. Subsequently, the plasmonic lenses were washed twice in a serum-free medium and added to the cells. The cells were then incubated for an additional 24 h, after which they were removed from the wells, and MTT was introduced to the cells. Following a 4 h incubation, the color of the MTT solution changed from yellow to purple due to the formation of formazan crystals in the living cells. The media containing MTT were then replaced with DMSO to dissolve the formazan crystals. Light absorption of each sample at a wavelength of 570 nm was measured by using a microplate reader (Tecan Trading AG, Switzerland). The percentage of viable cells was calculated by comparing the absorbance of the control cells (cells with medium only and no nanocomposite lens) with that of the cells incubated with the PCLs. The percentage of viable cells was determined by the formula: (*n*_cl_/*n*_c_) × 100, where *n*_cl_ represents the number of living cells in the plates containing the plasmonic lenses and MTT and *n*_c_ is the number of controlled living cells in plates containing MTT only.

## Results and Discussion

**Figure 1:**
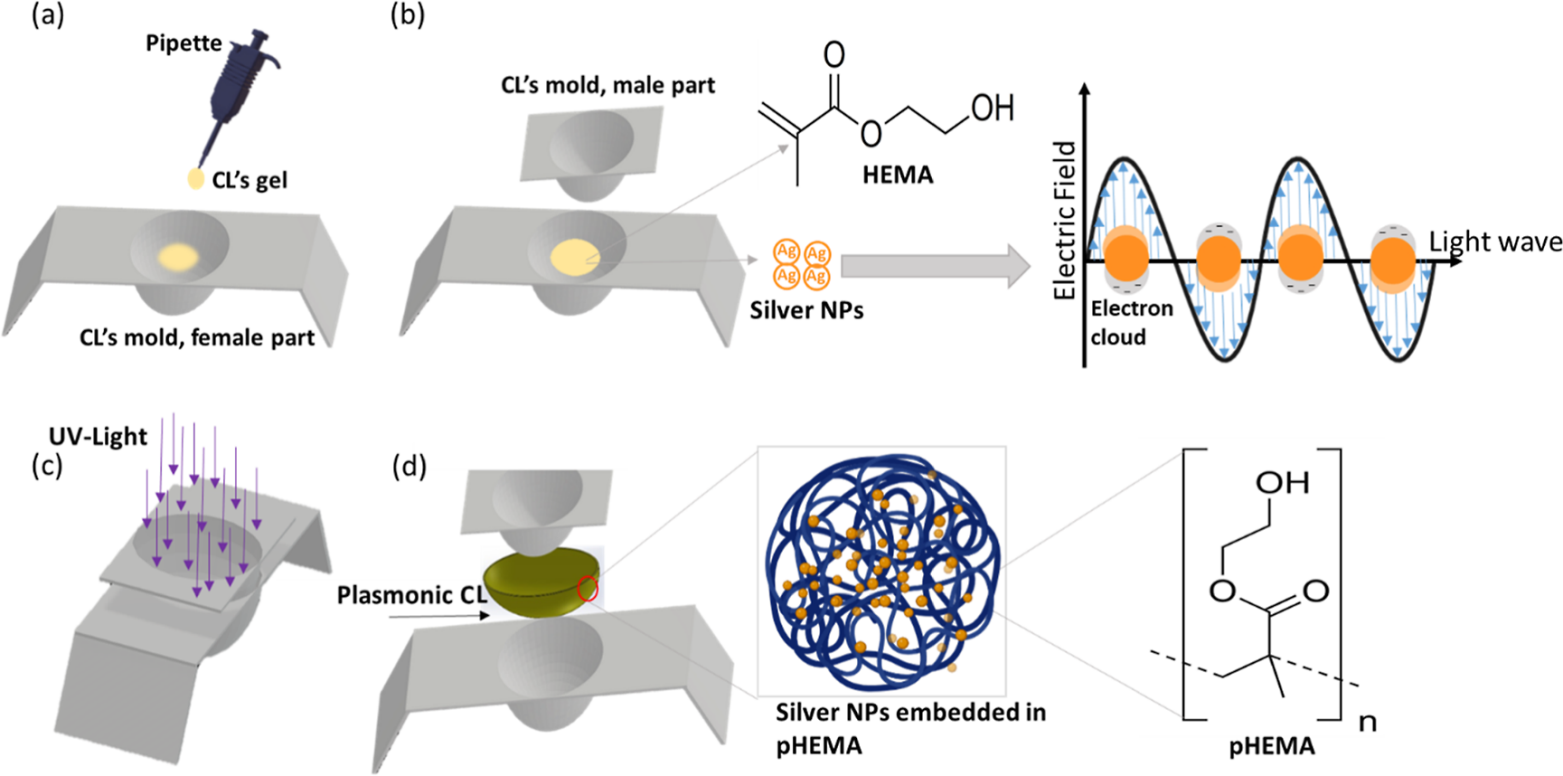
Overview of the fabrication of the PCLs and the working principle. (a) Dispensing the precursor gel into the contact lens mold. (b) The female part of the mold is overlaid with the male part, containing the key components: silver NPs and hydroxyethyl methacrylate (HEMA); the silver particles function to absorb blue light, constituting the working principle of the contact lens. (c) Exposing the mold to UV light (wavelength: 365 nm). (d) Removal of the male part of the mold, resulting in the extraction of the contact lens.

Silver nanoparticles exhibit remarkable light absorption efficiency due to their strong interaction with light through conduction electrons. When these electrons are excited by external light of a specific wavelength, they undergo collective oscillations known as surface plasmon (SP) oscillations.^24^ This phenomenon results in significant absorption of the resonant wavelength from the incident light (Figure 1).^25^ The resonant absorption wavelength of silver nanoparticles typically falls within the UV–vis light band; however, it can be finely tuned by manipulating the particles’ size, shape, and the local refractive index near the particle’s surface.^26−28^

In the development of blue light shielding lenses, spherical silver nanoparticles with sizes of 40 and 60 nm were embedded in custom-made soft contact lenses. Silver nanoparticles of these selected sizes offer a localized surface plasmon resonance (SPR) peak in the blue light region, allowing them to effectively absorb incident blue light (Figure 1).^29−32^ It is worth noting that the SPR peak position depends on the silver nanoclusters’ dielectric constant and the particles’ interdistance.^33−37^ The choice of spherical-shaped silver nanoparticles is deliberate as they present a single SPR peak. In contrast, asymmetrically shaped nanoparticles exhibit multiple absorption peaks.^38,39^ For instance, nanorods generate two SPR bands corresponding to their large and small axes.^40,41^

**Figure 2:**
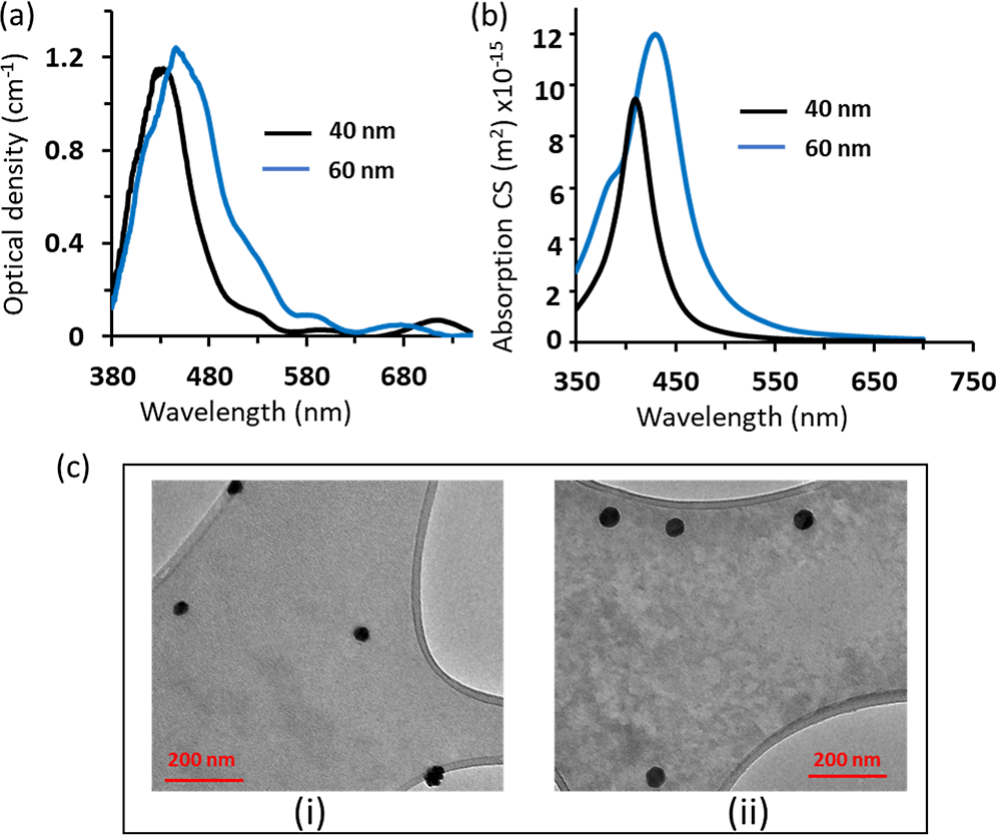
Light absorption and size characteristics of the employed silver nanoparticles. (a) Measurement of the optical density for the suspended silver nanoparticles with diameters of 40 (depicted in black) and 60 nm (depicted in blue). (b) Computed absorption cross-section of silver nanoparticles suspended in water with diameters of 40 and 60 nm. (c) Transmission electron microscopy (TEM) images illustrating the silver nanoparticles with diameters of 40 nm (i) and 60 nm (ii).

UV–vis spectroscopy measurements revealed that silver nanoparticles with sizes of 40 and 60 nm, suspended in an aqueous solution, exhibit SPR peaks in the blue light region. The SPR peaks were observed at wavelengths of 435 and 450 nm, respectively (Figure 2a). The observed shift in the positions of the SPR peaks for smaller and larger particles aligns with Mie theory predictions, indicating a red shift in the SPR peaks of silver nanoparticles as the particle size increases.^42^ The measured full width at half-maximum (fwhm) values of the resonance bands were 63 and 87 nm for silver particles of sizes 40 and 60 nm, respectively. Notably, as the particle size increased, the SPR peak red-shifted and broadened. Broadening of the fwhm with particle size is attributed to the retardation effects, as the incident electric field of the light cannot homogeneously polarize the large particles, resulting in exciting higher-order dipole modes.^43^ Additionally, large particles scatter light more efficiently, leading to additional broadening of the SPR peak due to irradiation damping. According to the Rayleigh model, the light absorption cross-section of a single nanoparticle is directly proportional to the third order of the particle’s diameters (*d*^3^), while its scattering cross-section is directly proportional to *d*^6^. Consequently, for small particles, light extinction is dominated by absorption, and for larger nanoparticles >50 nm, Rayleigh scattering dominates.^44^ Hence, silver nanoparticles with sizes larger than the chosen sizes, 40 and 60 nm, are expected to show broader resonance bands that may extend to block the desirable visible light range (510–780 nm).

Mie theory was used to model the light absorption for the suspended silver nanoparticles, and it was found that the SPR peaks were located at 413 and 434 nm for silver nanoparticles of sizes 40 and 60 nm, respectively (Figure 2b). Observably, SPR peaks for theoretical models were blue-shifted by 22 and 16 nm for particles of sizes 40 and 60 nm, respectively, as compared to the measured results. These discrepancies may be attributed to the particle–particle interactions, which are not considered in Mie theory, where the particle’s size and the local refractive index are the main parameters for predicting the extinction profile of the nanoparticles.^45^ Also, for the 60 nm particles, the modeling results showed a shoulder peak at 378 nm, attributed to the interband transitions.^46^ For silver nanoparticles, besides the SPR, there is a possibility of other electronic excitations. It is known that in metals, valence and conduction bands overlap; however, some inner energy levels do not split enough to overlap, and hence, the system may exhibit interband transitions similar to those in semiconductors.^47^ The interband transition peak was not observed for the particles of size 40 nm because it is expected to be located in the UV region (250 nm), a range not covered in the modeling.

The measured optical density (OD) at the resonance peaks for both suspended nanoparticle solutions was similar: 1.23 and 1.26 for particles of sizes 40 and 60 nm, respectively (Figure 2a). This confirms that both particle solutions have the same concentration as OD is correlated to mass or molar concentration and is directly proportional to the concentration.^48^ This justifies the similar maximum attenuation/absorption levels for both nanoparticle solutions. Sizes of the silver nanoparticles were measured by TEM, and the average sizes were 41.5 and 63.2 nm with standard deviations of 2.1 and 2.9 nm for the particles of sizes 40 and 60 nm, respectively (Figure 2c). The fine distribution of the nanoparticles’ size justifies the sharpness of the resonance peak (Figure 2a).

**Figure 3:**
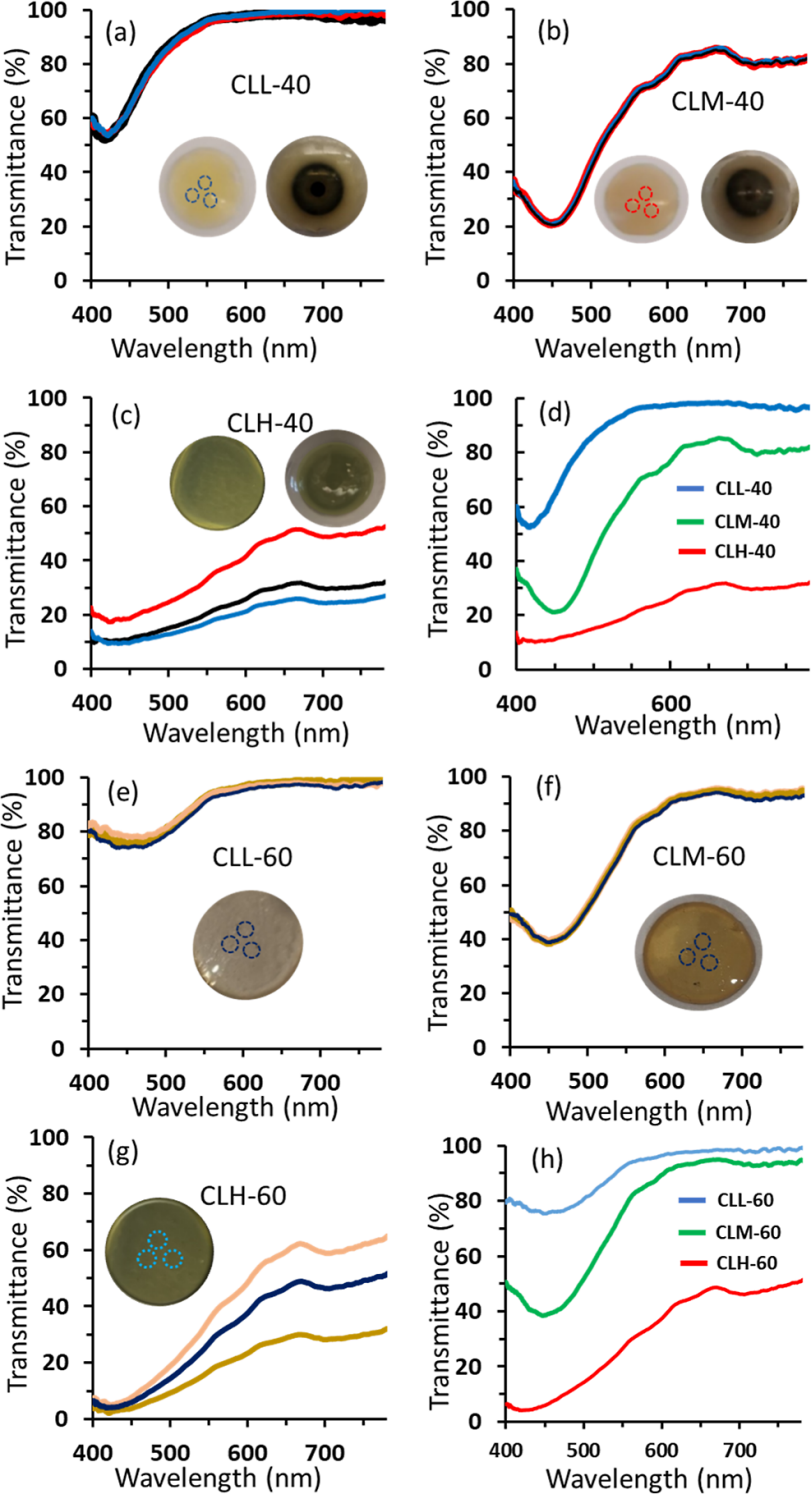
Transmittance analysis of the fabricated PCLs: (a–c) transmission spectra of PCLs loaded with 40 nm diameter silver nanoparticles at concentrations of 0.2 wt % (low), 0.4 wt % (medium), and 0.6% (high) denoted as CLL-40 (a), CLM-40 (b), and CLH-40 (c)—the inset displays photographs of the PCLs on an eye model and free-standing, with marked zones indicating where transmittance measurements were taken. (d) Transmission spectra of PCLs CLL-40, CLM-40, and CLH-40. (e–g) Transmission spectra of three PCLs containing 60 nm diameter silver nanoparticles at concentrations of 0.15 wt % (low), 0.35 wt % (medium), and 0.7% (high), labeled as CLL-60 (e), CLM-60 (f), and CLH-60 (g); the inset exhibits photographs of the PCLs, with marked zones indicating the locations of transmittance measurements. (h) Transmission spectra of the three PCLs loaded with 60 nm silver nanoparticles at varying concentrations.

PCLs were made of pHEMA-infused silver nanoparticles. Three different concentrations were prepared from the provided suspended silver nanoparticles of sizes 40 and 60 nm and were mixed with HEMA, which was transformed by UV-curing into PCLs, yielding six contact lenses with various concentrations of the silver nanoparticles. Three contact lenses out of the produced six lenses contained silver nanoparticles of size 40 nm made of the following concentrations: 0.2 wt % (low), 0.4 wt % (medium), and 0.6 wt % (high), denoted by CLL-40, CLM-40, and CLH-40, respectively. The remaining three contact lenses contained 60 nm silver nanoparticles with concentrations of 0.15 wt % (low), 0.35 wt % (medium), and 0.7 wt % (high), denoted by CLL-60, CLM-60, and CLH-60, respectively. The purpose of preparing PCLs with various concentrations of nanoparticles was to optimize the contact lenses’ performance. The main goal was to block the blue light range with an insignificant influence on the limpidity of the contact lens. The surface of each contact lens was scanned by a spectrophotometer attached to a microscope, and the transmission spectra of each lens was detected at three different sites located in the central zones (Figures 3, S1, and S2). Transmission spectroscopy was carried out to give indications about the distribution of the nanoparticles and to test the optical performance of the developed contact lenses. As theoretically predicted, the position of the SPR peak red-shifted with the particle’s size, and this behavior could be seen when comparing the transmission spectra of CLL-40 and CLL-60, which showed SPR peaks located at 420 and 450 nm, respectively (Figure 3a,e). Also, the SPR peak positions were found to red-shift with increasing concentration of the nanoparticles for the PCLs embedded silver nanoparticles of size 40 nm, CLM-40 (Figure 3a,b). The SPR peak was located at 420 nm for CLL-40 and shifted to 450 nm for CLM-40 (Figure 3d). This behavior might be attributed to the aggregation of the nanoparticles and/or the change in the effective refractive index. In contrast, there was no shift in the plasmonic peak position with increasing nanoparticle concentration of 60 nm size-loaded contact lenses, as the CLL-60 and CLM-60 showed the resonance peaks located at the same wavelength, 450 nm (Figure 3h). The shift in the SPR peak for increased concentration of 40 nm Ag NPs, but not 60 nm NPs, may indicate that the smaller nanoparticles are more prone to aggregation.

For PCLs of highly concentrated nanoparticles, CLH-40 and CLH-60, no sharp SPR peaks appeared, but instead, the light was attenuated over all the visible light band with extremely low transmittance in the blue light range (Figure 3c,g). This may be due to aggregation of nanoparticles into micrometer clusters, which do not support SPR and effectively scatter light.^45^ Increasing the nanoparticles’ concentration was found to be linked to the fwhm of the absorption/attenuation band. This was observable by comparing the transmission spectra of CLL-40 and CLM-40 (Figure 3d). Also, the discrepancy in the spectra of CLL-40 and CLL-60 is an example showing the broadening of the SPR band with particle size (Figure 3a,e). These results indicate that both particle size and concentration can customize the width of the absorption band. The sharp resonance dip observed in the transmission spectra of CLL-40 indicates that the nanoparticles were well dispersed in the contact lens hydrogel matrix (Figure 3a). The recorded optical responses showed insignificant differences in the transmission spectra measured at different sites in the central zones of the developed contact lenses, indicating the uniform distribution of the nanoparticles in the contact lenses embedded with low and medium concentrations of silver nanoparticles: CLL-40, CLM-40, CLL-60, and CLM-60 (Figure 3a,b,e,f). However, the contact lenses having high concentrations of the nanoparticles, i.e., CLH-40 and CLH-60, did not show sharp resonance absorption, and instead an attenuation for all visible light occurred with higher attenuation in the blue light region. Also, for heavily doped contact lenses (CLH-40 and CLH-60), significant differences in the transmission spectra were recorded at different regions inside the central zone, confirming the occurrence of huge aggregations (Figure 3c,g).

Overall, the transmission spectra showed that upon increasing the concentration of the nanoparticles, light absorption/attenuation (the dip band in the transmission spectra) increased—recording maxima of light blockage of 50, 80, and 92% for CLL-40, CLM-40, and CLH-40, respectively (Figure 3d). On the other hand, CLL-60, CLM-60, and CLH-60 filtered out up to 28, 65, and 98% of the incident blue light, respectively (Figure 3h). The average transmittance in the desirable light band (510–780 nm) and the undesirable/blocked light band (400–510 nm) were calculated based on the measured transmission data for judging the performance of the developed lenses. The average blue light protection for the six developed PCLs: CLL-40, CLM-40, CLH-40, CLL-60, CLM-60, and CLH-60 was 31, 71, 88.5, 22, 55, and 92%, respectively, and the mean transparencies in the desirable visible light range were 96, 78, 27, 96, 88, and 40.7%, respectively. In essence, there is a trade-off between the transparency of the contact lens and the level of protection against the blue light. Contact lenses CLH-40 and CLH-60 that provide high blue light protection (∼90%) showed low transmittance in the desirable region, and in contrast, the transparent contact lenses CLL-40 and CLL-60 showed a low level of protection against blue light. Therefore, for applications that require 90–100% blue light protection, developed PCLs based on silver nanoparticles are not recommended. However, for daily use basis, which requires protection in the range of 30–45% or even for protection ranges up to 60%, the developed PCLs: CLL-40, CLM-40, CLL-60, and CLM-60 showed competitive performance to that of the available spectacles in the market and superior efficiency compared to that of the previously reported BLCLs based on SiO_2_, ZnO, and TiO_2_. In a comparison of PCLs embedded with 40 and 60 nm size silver nanoparticles, those loaded with the 40 nm size are preferable as the absorption bands were narrower; thus, it did not extend to block the desirable passband. The best performances were achieved by CLL-40 and CLM-40 as they transmit the desirable band and highly block the blue light band. The ideal blue light protective lens is supposed to block 100% of the blue light and transmit 100% of the desirable light band (510–780 nm). Hence, the total score of the ideal lens is 200 points, resulting from the summation of the blue light protection score and the transmittance of the desirable band score. The closer the total score of the contact lens to 200, the better the performance. For low-Ag-loaded contact lenses: CLL-40 and CLL-60, the total scores of both were 127, and 122, respectively. Also, the medium-doped lenses, i.e., CLM-40 and CLM-60, had scores of 149 and 143, respectively. These results confirmed the better performance of the contact lenses loaded with the 40 nm silver nanoparticles, as aforementioned.

**Figure 4:**
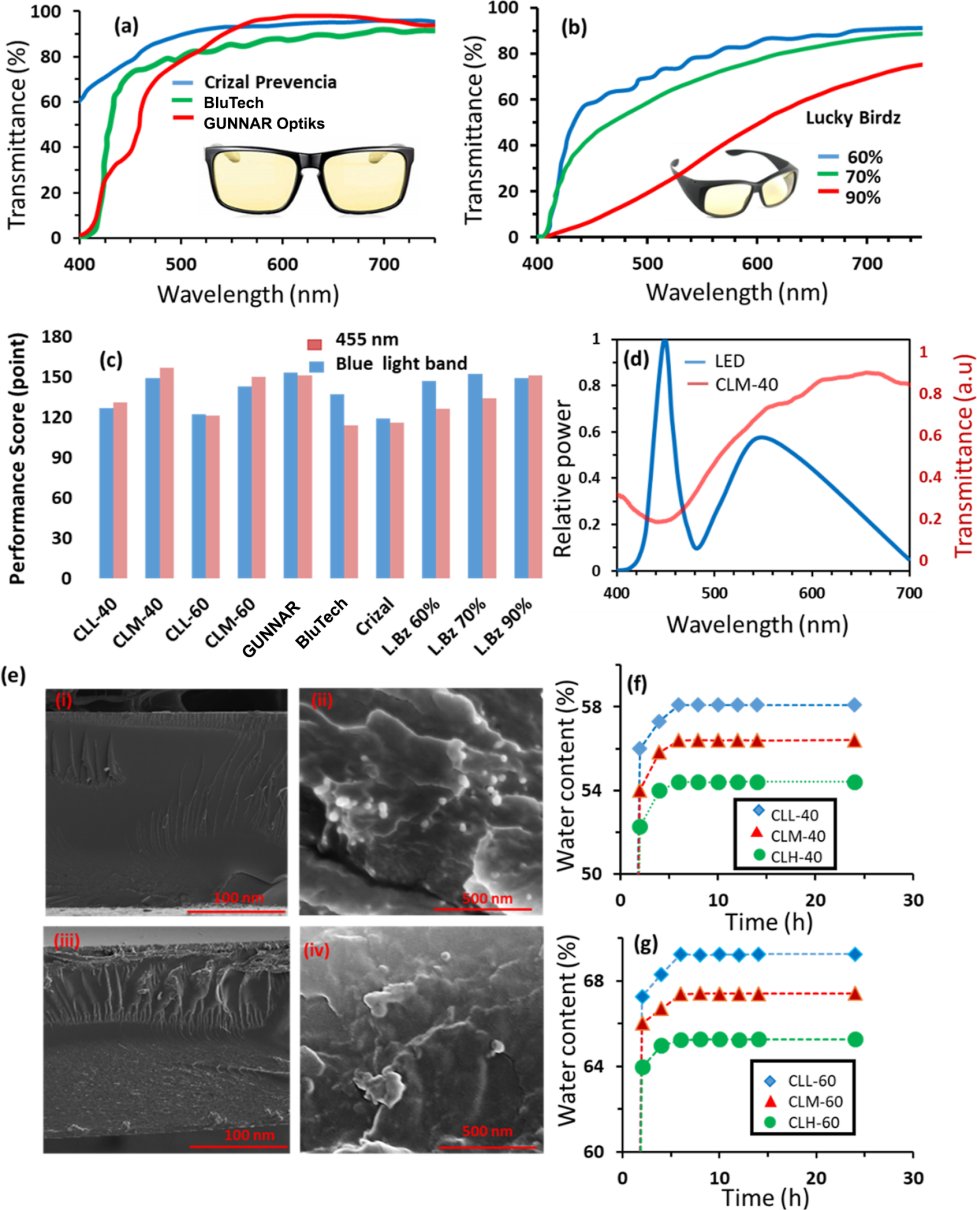
Optical characterization of commercial spectacles and the in-house-made contact lenses. (a) Transmission spectra of three commercial spectacles designed for blue light protection. (b) Transmission spectra for three distinct models of Lucky Birdz spectacles, each providing varying levels of blue light protection. (c) Comparative performance analysis between the developed PCLs and commercial spectacles. (d) Transmission spectra of the developed CLM-40 in response to the spectral power distribution emitted from LED screens. (e) Cross-sectional SEM images of the developed CLL-40, illustrating the distribution of 40 nm size nanoparticles at both low and high magnifications (i,ii) and SEM images of the cross-section of CLH-40 (iii,iv). (f) Water content of the PCLs: CLL-40, CLM-40, and CLH-40. (g) Water content of the PCLs: CLL-60, CLM-60, and CLH-60.

There are several blue light protective spectacles/glasses available in the market, including Caddis, BlueTech, LowBlueLights, LuckyBirdz Nectar, Crizal, and GUNNAR. These available products can block from 3% up to 100% of the blue light to suit the various needs. However, most of these available spectacles block up to 45%, which seems the favorable protection level for daily use, as exposure to some blue light is essential for good health. Studies reported that exposure to blue light during the daytime boosts alertness, helps memory and cognitive function, elevates mood, and regulates the circadian rhythm.^49^ Glasses that block 50–70% are recommended for individuals who suffer from light-induced migraines and for indoor activities such as using smartphones, TVs, and laptops. For sleep glasses, blocking up to 100% of the blue light is recommended. However, the color distortion is high for spectacle blocks above 60% of the blue light. Transmission spectra for some models of the commercial spectacles are displayed in Figure 4a,b. The displayed models showed mean blue light protection levels of 24, 49, and 59% for Crizal Prevencia, BluTech, and GUNNAR, respectively, and the mean transmittances in the passband were 95, 88, and 94%, respectively (Figure 4c). Comparing the performance of these three models, it was observed that GUNNAR performed the best in terms of clarity and protection as it blocks 59% of blue light and transmits 94% of the desirable visible light band with a total score of 153 out of 200 (Figure 4c). The BluTech model comes into the second rank with a total score of 137, and the Crizal model scored 119 out of 200. Three commercial models of the Lucky Birdz brand showed protection levels of 61, 72, and 90% accompanied by transmittances in the desirable band of 86, 80, and 59%, respectively—scoring 147, 152, and 149, respectively (Figure 4b,c). On the other hand, the developed PCLs showed superior performance to that of some available spectacles of the top brands. For instance, CLL-40 showed 31% protection and 96% transmittance with a total score of 127 outweighing Crizal Prevencia , which scored 119 resulting from 24% protection and 95% transmittance (Figure 4c). For medium blue light protection, the contact lenses CLM-40 and CLM-60, scored 149 and 143, respectively, compared to 153, 137, 147, and 152 for GUNNAR, BlueTech, Lucky Birdz 60%, and Lucky Birdz 70%, respectively (Figure 4c). Hence, the developed PCLs showed a very competitive performance (Figure 4c).

The blue light emitted from LED light sources and screens was found to have an intense spike at a wavelength of 455 nm (Figure 4d).^50^ Accordingly, wearing spectacles or contact lenses that show maximum protection at 455 nm may assist people, especially children, in avoiding complications resulting from blue light exposure. Advantageously, the developed lenses CLM-40 and CLM-60 showed spike protection at the intense spike wavelength emitted from LED screens and digital devices (Figure 4d). The contact lenses CLM-40 and CLM-60 provided high protection levels of 79 and 62% at the screen spike emitted wavelength of 455 nm, with transmittances of 78 and 88% in the desirable region, respectively. In contrast, none of the available spectacles show spike protection at the wavelength 455 nm (Figure 4a,b). Having a spike protection at 455 nm allows a high level of protection from indoor blue light, and at the same time, it provides clear vision by allowing the desirable light band to pass through the lenses. At the intense spike of wavelength 455 nm, the protections of the low and highly doped contact lenses, i.e., CLL-40, CLH-40, CLL-60, and CLH-60 were found to be 35, 89, 25, and 93%, respectively, and among the six developed PCLs, CLL-40, CLM-40, and CLM-60 are highly recommended for indoor activities for their superior performance (Figure 4c). BluTech proclaimed that the lenses that provide blue light blockage up to 59% for 455 nm improve sleep, reduce digital eye strain, and improve productivity. The protection of the presented six commercial spectacle models at the wavelength of 455 nm was recorded to be 21, 57, 26, 40, 54, and 92% for Crizal Prevencia, GUNNAR, BluTech, Lucky Birdz 60%, Lucky Birdz 70%, and Lucky Birdz 90%, respectively (Figure 4c). The developed lenses: CLL-40, CLM-40, and CLM-60 showed better protection at the intense spike wavelength (455 nm) of the LED screens as they provided higher blue light shielding and transmittance scoring of 131, 157, and 150, respectively, compared to 116, 151, 114, 126, 134, and 151 for Crizal Prevencia, GUNNAR, BluTech, Luck Birdz 60%, Lucky Birdz 70%, and Lucky Birdz 90%, respectively (Figure 4c). The developed CLM-40 proved to be the best among all samples and the commercial spectacles as it scored 157 points compared to 151 scored by the best-performing spectacle, GUNNAR. The superior performance of the developed lens is attributed to the resonance absorption of the silver nanoparticles at 455 nm. Hence, according to the provided figures, the developed lenses outweighed the performance of the available spectacles when it comes to protection against the emitted light of screens and digital devices.

To investigate the aggregation of nanoparticles within the developed lenses, we utilized SEM to examine cross sections of the lowest- and highest-loaded contact lenses, namely, CLL-40 and CLH-40 (Figure 4e). The images revealed substantial aggregations in the microscale for CLH-40, whereas CLL-40 exhibited fewer clusters in the nanoscale, with the majority of particles dispersed individually. These findings provide insights into the absence of the SPR dip observed in the transmission spectra of highly concentrated nanoparticle-infused contact lenses (CLH-40 and CLH-60).

Maintaining appropriate levels of water retention in contact lenses is crucial for wearer comfort and to prevent issues such as eye irritation, itching, burning, and a stinging sensation.^51^ To assess the impact of nanoparticles on the “wetness” level, we measured the water retention of the developed PCLs. Additionally, water content serves as an indicator of oxygen permeability, vital for the cornea, which lacks its own blood vessels and relies on airborne oxygen.^52^ As anticipated, the water content of the developed lenses decreased with an increasing nanoparticle concentration, irrespective of the particle size used (Figure 4f,g). This reduction in water content may be attributed to nanoparticles occupying space between polymeric chains, thereby diminishing the effective pore size and reducing water storage spaces in the hydrogel matrix. The water content percentages for low-, medium-, and high-nanoparticle-doped contact lenses (CLL-40, CLM-40, and CLH-40) were 58.1, 56.4, and 54.4%, respectively (Figure 4f). In contrast, for CLL-60, CLM-60, and CLH-60, the water contents were 69.3, 67.4, and 65.3%, respectively (Figure 4g). It is noteworthy that the water content of commercial soft contact lenses typically ranges from 38 to 79%, with values below 45% considered low and those above 45% considered high according to the Food and Drug Administration (FDA) classification for contact lenses.^51,53^ All developed PCLs exhibited a high level of water content comparable to that of commercial contact lenses. Notably, lenses loaded with 60 nm silver nanoparticles showed a higher water content at all concentration levels (low, medium, and high) compared to their counterparts loaded with 40 nm silver nanoparticles. This phenomenon could be attributed to larger nanoparticles (60 nm) disrupting the polymer chain and leaving a larger effective size in the hydrogel matrix.^54^ Despite changes in nanoparticle concentration from low to high inducing water content changes of 3.7 and 4% for PCLs loaded with 40 and 60 nm silver nanoparticles, respectively, these alterations were relatively minor. Particularly, at medium concentrations of nanoparticles (0.2% and 0.35 wt %), a reduction of 1.7 and 1.9% was observed for contact lenses embedded with 40 and 60 nm, respectively, suggesting an insignificant impact on the wetness of the developed PCLs. The time required for saturated swelling/full hydration remained unaffected by particle concentration and size (Figure 4f,g), with each of the six developed PCLs reaching full hydration within approximately 8 h. In summary, low- and medium-loaded PCLs are recommended for blue light protection based on their intriguing optical performance. However, highly nanoparticle-doped lenses such as CLH-40 and CLH-60 are excluded due to significant nanoparticle aggregation.

**Figure 5:**
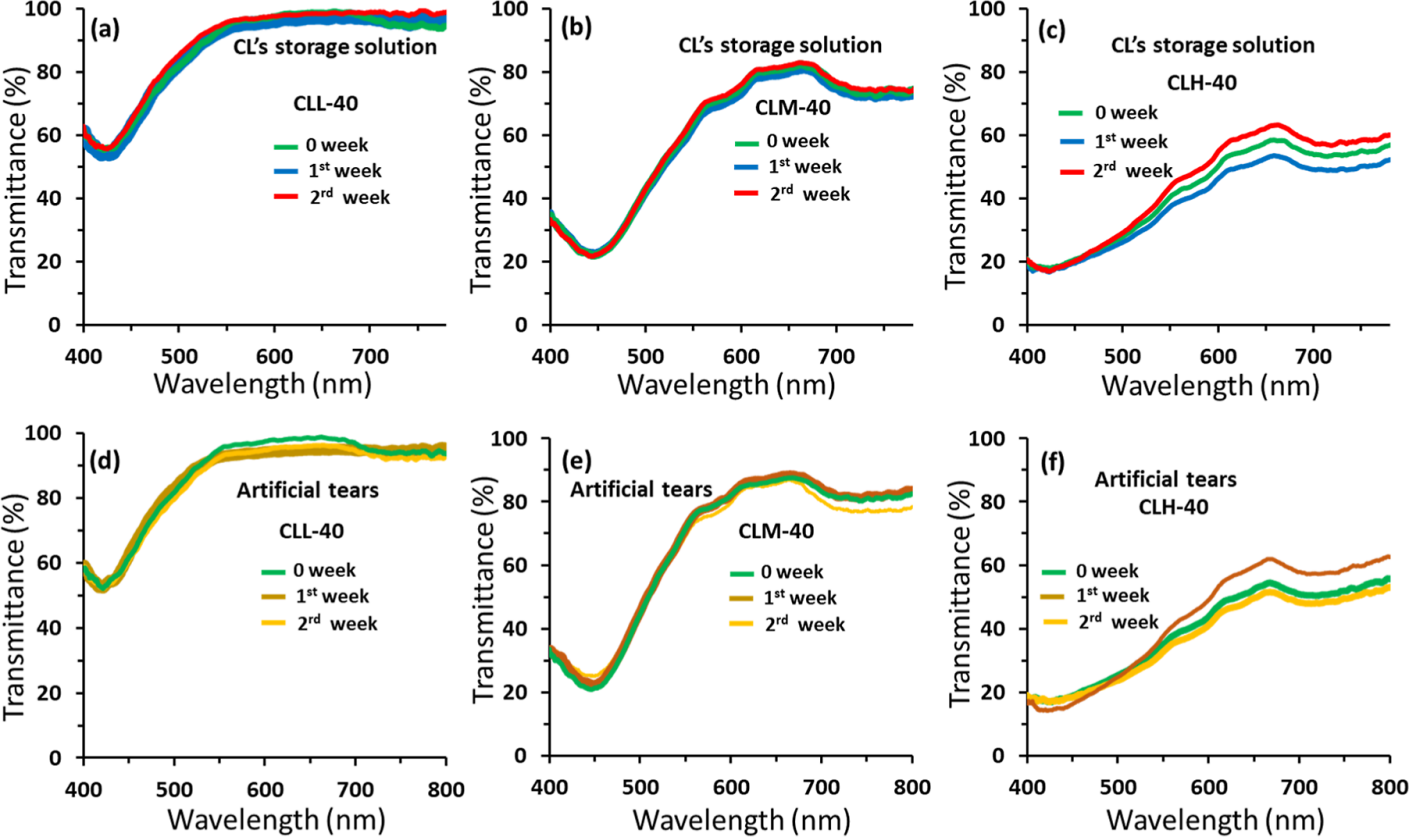
Leakage test for the contact lens-embedded silver nanoparticles with a diameter of 40 nm: CLL-40, CLM-40, and CLH-40. (a–c) Transmission spectra of the contact lenses stored in contact lens’s storage solutions: (a) CLL-40, (b) CLM-40, and (c) CLH-40. (d–f) Transmission spectra of the contact lenses stored in artificial tears: (d) CLL-40, (e) CLM-40, and (f) CLH-40.

**Figure 6:**
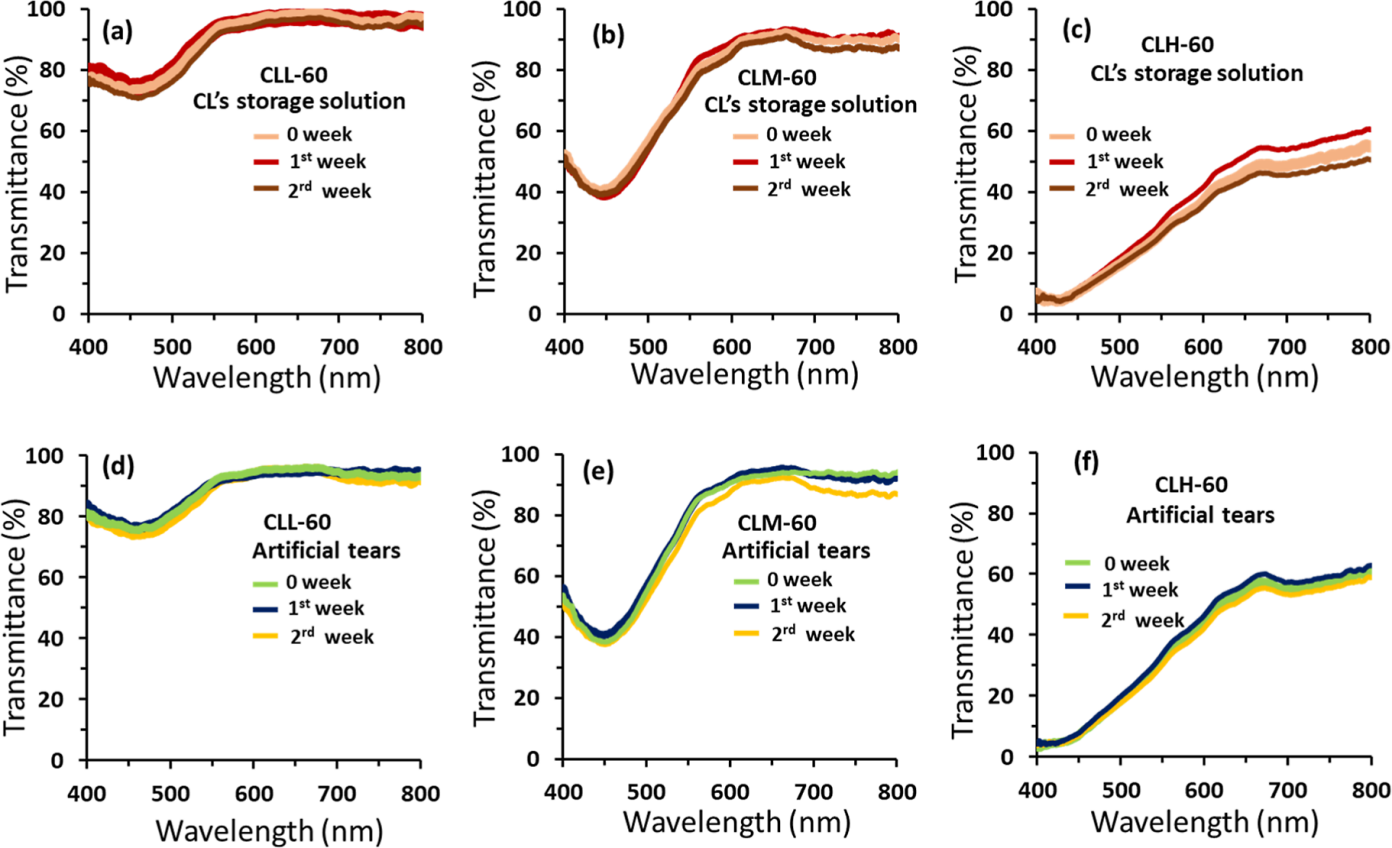
Leakage test for contact lens-embedded silver nanoparticles (60 nm): CLL-60, CLM-60, and CLH-60. (a–c) Transmission spectra of the contact lenses stored in contact lens’s storage solutions: (a) CLL-60, (b) CLM-60, and (c) CLH-60. (d–f) Transmission spectra of the contact lenses stored in artificial tears: (d) CLL-60, (e) CLM-60, and (f) CLH-60.

Ensuring the stable performance of PCLs in tears is of utmost importance, with the contact lens storage solution being expected to exert no adverse effects on performance. The stability of the developed PCLs was systematically assessed by subjecting them to a lens storage solution and artificial tears, ensuring the nanoparticles remained securely embedded in the hydrogel matrix without any leakage. Transmission spectra were continuously monitored over time following exposure to these solutions, with a decline in the resonance absorption peak, manifested as a dip in the transmission spectra, serving as an indicator of potential nanoparticle leakage. The contact lenses’ transmission spectra were initially recorded, and subsequently, the samples were immersed in the storage solution for 2 weeks, with weekly transmission spectra recordings. The recorded spectra were then analyzed to detect any signs of leakage (Figures 5 and 6). For low- and medium-nanoparticle-doped lenses (CLL-40, CLM-40, CLL-60, and CLM-60), consistent and insignificant changes were observed over time, possibly attributable to random errors (Figures 5 and 6). Importantly, the protective capabilities of these lenses remained constant. In contrast, contact lenses with high nanoparticle doping, specifically CLH-40 and CLH-60, exhibited significant changes in their transmission spectra. This may be attributed to potential slight shifts in the measuring site exacerbated by the high aggregation observed in these lenses, leading to variations in transmittance across the lens surface (Figures 5c and 6c). To further evaluate performance stability, samples were immersed in a storage solution for 2 weeks, followed by exposure to an artificial tear solution for an additional 2 weeks, totaling 4 weeks (Figures 5d–f and 6d–f). The results affirm the effective entrapment of nanoparticles within the hydrogel matrix, as no signs of leakage were detected throughout the entire 4 week duration, validating the robust stability of the developed PCLs.

**Figure 7:**
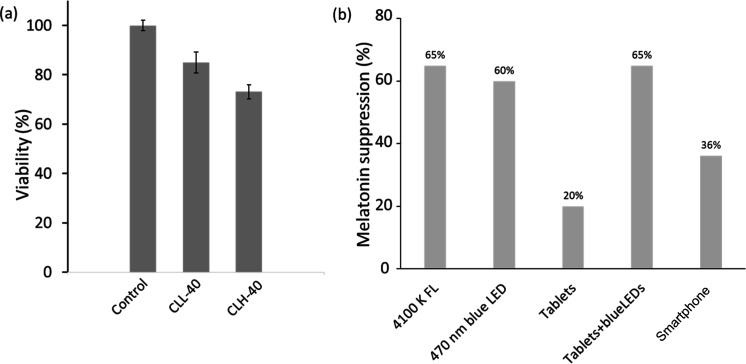
Cytotoxicity assessment of PCLs. (a) MTT cytotoxicity test conducted on RAW 264.7 model cells for the developed contact lenses, namely, CLL-40 and CLH-40. (b) Levels of melatonin suppression resulting from exposure to different artificial light sources.

Given that the developed lenses come into contact with human cells, an MTT assay was employed to evaluate their potential toxicity to RAW 264.7 cell culture over a 24 h period (Figure 7a). Previous studies on pHEMA CLs stored in phosphate buffer solution indicated cellular viability comparable to that of control cells.^55^ However, a reduction in cell viability, notably 15 and 27%, was observed for lenses CLL-40 and CLH-40, respectively. The lens with fewer nanoparticles exhibited improved cell availability, possibly due to the antimicrobial properties of silver nanoparticles being outweighed by their relative cytotoxicity. A similar effect was noted when pHEMA CLs were stored in antimicrobial and borate-buffered solutions, leading to a decrease of over 20% in cell viability.^55^ Thus, the concentration of silver nanoparticles may be linked to the observed cytotoxic effects, necessitating further studies to optimize the nanoparticle concentration and eliminate toxicity without compromising the blue light protection efficiency of the developed PCLs.

In our contemporary, digitalized world, avoiding blue light in the evening proves to be challenging and inconvenient. Exposure to light from self-luminous, blue light-emitting displays before bedtime is associated with sleep disorders, primarily due to the suppression of nocturnal melatonin (Figure 7b).^56^ At nighttime, a reduction of the nocturnal melatonin levels by up to 36.1% was reported when an individual was exposed to smartphone displays (LCD, AMOLED) that had a circadian illuminance of 105.2 biolux at a distance of 20 cm from the eyes (Figure 7b).^1^ Sitting in rooms illuminated by a 4100 K full-spectrum fluorescent lamp (4100 K FL) can deliver circadian illuminance up to 800 biolux, which suppresses melatonin secretion by 65% (Figure 7b).^1^ Nighttime exposure to the 470 nm blue LED was found to suppress melatonin by 60% (Figure 7b).^1^ Furthermore, a study carried out on 13 volunteers found that the nocturnal melatonin was suppressed by 20 and 65% due to exposure of the participants to tablets’ screens and tablets along with blue LEDs, respectively, for 2 h (Figure 7b).^56^ Blocking blue light using blue light protective lenses a few hours before bedtime may mitigate these effects by maintaining circadian illuminance below the threshold level (15 biolux), at which point impacts on melatonin secretion become negligible.^1^

PCLs can serve as an effective ophthalmic device for blue light protection, operating based on the localized SPR of silver nanoparticles in the blue light region of the spectrum. Unlike dye-tinted lenses susceptible to photodegradation or photobleaching, silver nanoparticle-embedded lenses offer a robust solution without complex fabrication methods, making large-scale production feasible.^57,58^ While other techniques, such as Bragg mirrors, can be designed to filter out blue light, applying them to contact lenses presents significant challenges due to the weak mechanical and thermal properties of the lens material. The simplicity and effectiveness of silver nanoparticle-embedded lenses make them a promising option for practical and scalable blue light protection.

## Conclusions

Plasmonic soft contact lenses incorporating silver nanoparticles have been successfully engineered to mitigate the impact of blue light exposure. These lenses exhibit high transparency in the passband, offering varying levels of blue light protection depending on the concentration of the embedded nanoparticles. Notably, these lenses demonstrate a pronounced protective peak at a wavelength of 455 nm, targeting the intense blue light emitted by commercial displays. To assess biocompatibility, the cytotoxicity of the developed lenses was evaluated using the MTT assay on RAW265.6 model cells. Results indicated overall biocompatibility except for lenses with high nanoparticle concentrations (CLH-40 and CLH-60), which exhibited a 27% reduction in cell viability. Conversely, the low-concentration nanoparticle lens (CLL-40) maintained a cell viability at 85%. In addition to their protective features, the water retention of the lenses is comparable to that of FDA-classified high-water-content contact lenses. The impregnated silver nanoparticles within the pHEMA hydrogel matrix minimally impact water retention. Stability tests conducted over 1 month in artificial tears and contact lens storage solutions demonstrated consistent optical performance. These nanoparticle-infused contact lenses represent a significant advancement in the development of effective and scalable blue light filtering devices. Their potential to prevent the suppression of nocturnal melatonin secretion positions them as valuable tools for promoting balanced sleep for individuals.

## Supporting Information Available

Distribution of light intensity transmitted through the plasmonic contact lenses loaded with silver particles (PDF)
