# Supplementary material for: Plasmonic Contact Lenses Based on Silver Nanoparticles for Blue Light Protection
Source: ACS Appl Nano Mater. 2024 Mar 6;7(6):5956–66. doi: 10.1021/acsanm.3c05857 (PMC10964193; doi:10.1021/acsanm.3c05857)
Supplement: Supplementary file 1 — an3c05857_si_001.pdf [file an3c05857_si_001.pdf]

Supporting Information

Plasmonic Contact Lenses Based on Silver Nanoparticles for Blue Light Protection

Mohamed Elsherif[a,b], Ahmed E. Salih[a], Fahad Alam[a], Ali K. Yetisen[c], Khalil B. Ramadi[b,d], Haider Butt[a]**

[a]Department of Mechanical Engineering, Khalifa University, Abu Dhabi 17788, UAE

[b]Division of Engineering, New York University Abu Dhabi, Abu Dhabi 129188, UAE

[c]Department of Chemical Engineering, Imperial College London, London SW7 2AZ, UK

[d]Tandon School of Engineering, New York University, NY 11201, USA

*correspondence emails: M.E: elsherifmohamed109@gmail.com, H.B: haider.butt@ku.ac.ae

Keywords: contact lenses; biomaterials; blue light filtering; silver nanoparticles; pHEMA

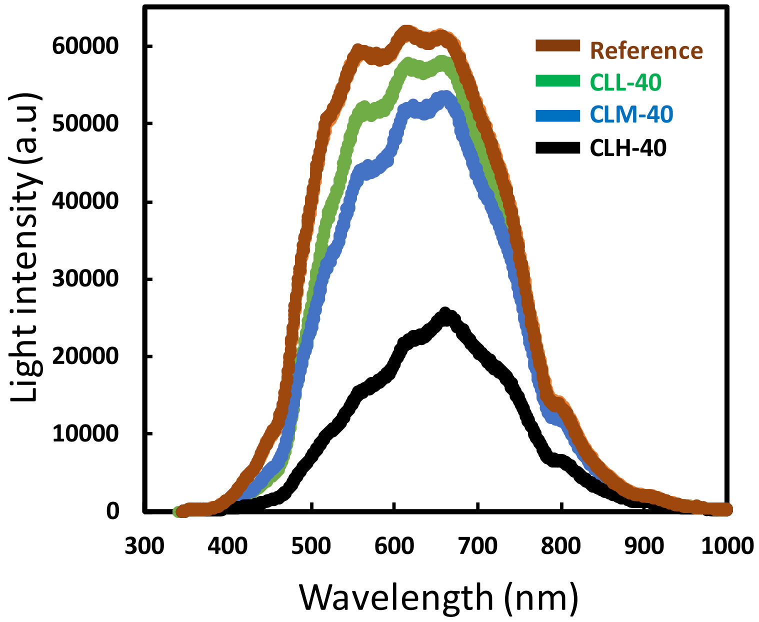

Figure S1. The light intensity distribution of the light source transmitted through the developed plasmonic contact lenses embedded with silver nanoparticles of size 40 nm.

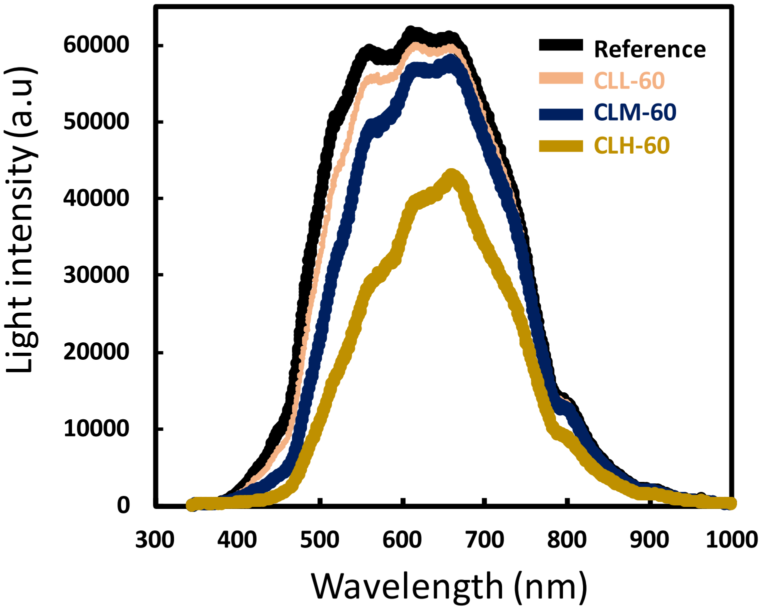

Figure S2. The light intensity distribution of the light source transmitted through the developed plasmonic contact lenses embedded with silver nanoparticles of size 60 nm.
